# “Work‐to‐Work” exercise slows pulmonary oxygen uptake kinetics, decreases critical power, and increases W’ during supine cycling

**DOI:** 10.14814/phy2.13916

**Published:** 2018-11-13

**Authors:** Richie P. Goulding, Denise M. Roche, Simon Marwood

**Affiliations:** ^1^ School of Health Sciences Liverpool Hope University Liverpool United Kingdom

**Keywords:** Critical power, exercise tolerance, oxidative metabolism, oxygen uptake kinetics, power–duration relationship, work‐to‐work exercise

## Abstract

We have previously demonstrated that the phase II time constant of pulmonary oxygen uptake kinetics ($\tau_{\dot{v}o_{2}}$) is an independent determinant of critical power (CP) when O_2_ availability is not limiting, that is, during upright cycle exercise in young, healthy individuals. Whether this causative relationship remains when O_2_ availability is impaired remains unknown. During supine exercise, which causes an O_2_ availability limitation during the exercise transition, we therefore determined the impact of a raised baseline work rate on $\tau_{\dot{v}o_{2}}$ and CP. CP, $\tau_{\dot{v}o_{2}}$, and muscle oxygenation status (the latter via near‐infrared spectroscopy) were determined via four severe‐intensity constant‐power exercise tests completed in two conditions: (1) with exercise initiated from an unloaded cycling baseline (U→S), and (2) with exercise initiated from a moderate‐intensity baseline work rate of 90% of the gas exchange threshold (M→S). In M→S, critical power was lower (U→S = 146 ± 39 W vs. M→S = 132 ± 33 W, *P *=* *0.023) and $\tau_{\dot{v}o_{2}}$ was greater (U→S = 45 ± 16 sec, vs. M→S = 69 ± 129 sec, *P *=* *0.001) when compared to U→S. There was no difference in tissue oxyhemoglobin concentration ([HbO_2_ + MbO_2_]) at baseline or during exercise. The concomitant increase in $\tau_{\dot{v}o_{2}}$ and reduction in CP during M→S compared to U→S shows for the first time that $\tau_{\dot{v}o_{2}}$ is an independent determinant of CP in conditions where O_2_ availability is limiting.

## Introduction

Tolerance to whole‐body, high‐intensity exercise is a major determinant of quality of life, mortality, and exercise performance (Myers et al. 2002). Understanding the physiological antecedents of high‐intensity exercise tolerance therefore remains an essential goal for the development of optimal therapeutic interventions aimed at enhancing exercise tolerance in both patient and athletic populations. High‐intensity exercise tolerance for durations of ~2–30 min is well characterized by a hyperbolic relationship between external work rate and the tolerable duration of exercise, known as the power–duration relationship (Monod and Scherrer 1965; Moritani et al. 1981; Jones et al. 2008). The asymptote of the power–duration relationship has been termed critical power, a notionally sustainable work rate (Moritani et al. 1981), with the fixed and finite volume of work available during exercise above critical power known as *W*’ (Fukuba et al. 2003). The crucial role of critical power in determining high‐intensity exercise tolerance is underlined by critical power representing a physiological threshold above which pulmonary oxygen uptake ($\dot{V}O_{2}$), blood acid–base balance, and concentrations of intramuscular metabolites cannot be stabilized (Poole et al. 1988; Jones et al. 2008; Vanhatalo et al. 2016). The limit of exercise tolerance above critical power is thus associated with the attainment of a critical muscle metabolic milieu of low [phosphocreatine] ([PCr]) and pH, and high [inorganic phosphate] ([Pi]) (Vanhatalo et al. 2010). Hence, critical power represents the functional expression of an underlying “critical $\dot{V}O_{2}$” (Poole et al. 2016) which could be realized in other forms of exercise, such as walking and running, with a resultant critical speed. Any activity of everyday living that requires a metabolic rate in excess of the critical $\dot{V}O_{2}$ is therefore predictably unsustainable in accordance with the parameters of the power–duration relationship and their physiological consequences. The physiological determinants of critical power and *W*’ are therefore those that govern the tolerable duration of high‐intensity exercise and need fully identifying. These determinants, however, remain incompletely understood.

Recent evidence has implicated the time constant of the fundamental phase of pulmonary $\dot{V}O_{2}$ kinetics ($\tau_{\dot{v}o_{2}}$) as a determinant of critical power (Murgatroyd et al. 2011; Goulding et al. 2017, 2018). In particular, we showed a concomitant increase in $\tau_{\dot{v}o_{2}}$ (i.e., $\dot{V}O_{2}$ kinetics were slower) alongside a reduction in critical power during exercise initiated from a raised metabolic baseline in upright cycle exercise in young healthy adults (Goulding et al. 2018), suggesting that $\tau_{\dot{v}o_{2}}$ is an independent determinant of critical power. Prior to this, we also showed that a bout of high‐intensity “priming” exercise decreased $\tau_{\dot{v}o_{2}}$ and increased critical power during supine exercise (Goulding et al. 2017). However, unlike in the upright position, exercise in the supine position results in the loss of a hydrostatic gradient, which impairs muscle perfusion pressure and slows pulmonary $\dot{V}O_{2}$ kinetics (Hughson et al. 1993; MacDonald et al. 1998; Koga et al. 1999; Jones et al. 2006). Hence, the improved critical power following priming exercise in this prior study (Goulding et al. 2017) may therefore have been due to the improved O_2_ availability that attended priming exercise, rather than the concomitant faster $\dot{V}O_{2}$ kinetics per se. Indeed, the inverse relationship between $\tau_{\dot{v}o_{2}}$ and critical power that we (Goulding et al. 2017, 2018) and others (Murgatroyd et al. 2011) have observed in the upright position was absent during supine exercise. Taken together, these data suggest that when O_2_ availability is limiting to $\tau_{\dot{v}o_{2}}$ (such as during supine exercise), O_2_ delivery, rather than $\tau_{\dot{v}o_{2}}$, primarily determines critical power. This possibility warrants further investigation in order to provide a more complete picture of the relationship between $\tau_{\dot{v}o_{2}}$ and critical power, and will have particular relevance for a number of clinical populations that possess slow $\dot{V}O_{2}$ kinetics due to impaired O_2_ delivery rather than intrinsic mitochondrial inertia (Poole and Jones 2012; Hirai et al. 2015).

$\tau_{\dot{v}o_{2}}$ is greater when exercise transitions are initiated from an elevated metabolic rate (“work‐to‐work” exercise) compared to that associated with a baseline of unloaded cycling (Brittain et al. 2001; Wilkerson and Jones 2006; Breese et al. 2013; Goulding et al. 2018), an effect that is also apparent during supine exercise (DiMenna et al. 2010b). This slowing of the $\dot{V}O_{2}$ kinetics with work‐to‐work exercise appears to occur without any meaningful alterations to O_2_ availability (Bowen et al. 2011; Wüst et al. 2014). Hence, in the supine position, work‐to‐work exercise versus exercise transitions performed from an unloaded baseline, can be utilized as a means by which to test the hypothesis that $\tau_{\dot{v}o_{2}}$ is not a determining factor of critical power when O_2_ delivery is limiting to $\tau_{\dot{v}o_{2}}$.

The purpose of this investigation was therefore to assess the effect of work‐to‐work exercise on pulmonary $\dot{V}O_{2}$ kinetics and critical power during supine exercise, the latter imposing an O_2_ delivery limitation in both conditions. We hypothesized that work‐to‐work exercise, when compared to an unloaded baseline, would result in (1) a greater $\tau_{\dot{v}o_{2}}$ and (2) no change in critical power. Realization of these hypotheses would inculpate O_2_ availability, and not $\tau_{\dot{v}o_{2}}$_,_ as the key determinant of critical power where there is an insufficiency of O_2_ availability.

## Materials & Methods

### Participants

Eight healthy male participants (mean ± SD, age =26 ± 4 years; height = 179 ± 9 cm; mass = 81 ± 7 kg), none of whom were highly trained, provided written informed consent to participate. The study was conducted in accordance with the Declaration of Helsinki, and had been approved by the Liverpool Hope University Research Ethics Committee. Benefits and risks of the study, as well as rights to withdrawal and confidentiality were explained to each participant prior to participation. Participants were instructed not to consume caffeine or alcohol within the preceding 3 h and 24 h prior to each visit, respectively. Participants were also instructed not to perform heavy exercise within the preceding 24 h prior to each visit and to arrive 3 h postprandial.

### Procedures

All testing took place in a well‐ventilated laboratory that was kept between temperatures of 18–21°C. Participants attended the laboratory on nine separate occasions over a 3‐ to 6‐week period. Each test was scheduled at the same time of day (±2 h) and with a minimum of 24 h separating each test. Participants completed one preliminary trial and eight experimental trials. All tests were performed using an electronically braked ergometric unit (Lode Angio, Groningen, The Netherlands), while lying flat on an Echo Cardiac Stress Table (Lode, Groningen, The Netherlands). Hand rails were available for participants to grip throughout the tests to minimize backward movement when forces were applied to the pedals, and an adjustable shoulder pad was positioned above the participant's shoulder to further prevent backward movements. Participant's feet were strapped securely to the pedals. The position of the shoulder pad and the distance between the hip and the crank was recorded and replicated during each visit. Throughout all exercise tests, participants were instructed to maintain a cadence of 80 rev/min, and exhaustion was defined as when the participant's cadence dropped below 70 rev/min. Time to exhaustion was measured to the nearest second in all tests.

### Preliminary trial

Participant's height and weight were recorded, after which each participant undertook an incremental ramp test until the limit of tolerance to determine $\dot{V}O_{2}\text{max}$, the gas exchange threshold (GET), and the power outputs for subsequent tests. The incremental ramp test consisted of 3 min of baseline pedaling at 30 W, followed by a linear increase in power output of 25–30 W min^−1^ (the ramp rate was selected based on participant's self‐reported exercise history) as a smooth function of time until the limit of tolerance was reached. Ventilatory and gas exchange variables were measured continuously breath by breath throughout each text. $\dot{V}O_{2}\text{max}$ was defined as the highest $\dot{V}O_{2}$ value measured over 30 sec. The GET was taken as a noninvasive estimate of the lactate threshold using various previously established criteria; including (1) a disproportionate rise in CO_2_ production ($\dot{V}{\text{CO}}_{2}$) relative to $\dot{V}O_{2}$; (2) an increase in minute ventilation ($\dot{V}E$) relative to $\dot{V}O_{2}$ ($\dot{V}E$/$\dot{V}O_{2}$) without an increase in $\dot{V}E$ relative to $\dot{V}{\text{CO}}_{2}$ ($\dot{V}E$/$\dot{V}{\text{CO}}_{2}$); and (3) an increase in end‐tidal O_2_ tension without decreasing end‐tidal CO_2_ tension. The mean response time (MRT) of $\dot{V}O_{2}$ during ramp exercise was taken as the time between the onset of the ramp test and the intersection between baseline $\dot{V}O_{2}$ ($\dot{V}O_{2b}$) and backward extrapolation of the regression line of the $\dot{V}O_{2}$–time relationship. The $\dot{V}O_{2}$–time relationship was determined as previously described (Boone et al. 2008). Work rates for subsequent tests were therefore calculated using the linear regression line of the $\dot{V}O_{2}$–time relationship and solving for $\dot{V}O_{2}$, with account taken of the MRT.

### Experimental trials

The following eight visits required participants to exercise to exhaustion at four fixed severe‐intensity constant‐power outputs, each performed on two occasions; on four occasions the transition to these severe‐intensity power outputs was made from a baseline of unloaded cycling (U→S), and on the other four occasions the transition to severe exercise was made from a baseline of moderate‐intensity cycling at 90% of the GET (M→S). The power outputs for the severe‐intensity bouts of exercise were chosen on the basis of performance in the preliminary trial and were calculated to be in the range 50%∆ (i.e., a work rate calculated to require 50% of the difference between the GET and $\dot{V}O_{2}\text{max}$) to 110% $\dot{V}O_{2}\text{max}$. The power outputs were selected to produce a range of exercise tolerance times spanning 2–15 min, with at least 5 min between the longest and shortest tests (Hill 1993). On occasions where a particular power output produced an exercise tolerance time outside of this desired range, the power output was adjusted and the test was repeated on a separate occasion. These power outputs are subsequently referred to as WR1, WR 2, WR 3, and WR 4, with WR 1 being the lowest and WR 4 being the highest power outputs, respectively. The power outputs were presented in random order, whereas participants alternated between M→S and U→S to prevent an order effect from occurring. Participants were not informed of their work rate or performance until the entire project had been completed. U→S consisted of 3 min of unloaded baseline pedaling at 0 W, following which a step increase in power output was applied to the required severe‐intensity work rate, and participants exercised until the limit of tolerance. In M→S, participants performed 3 min of unloaded baseline pedaling at 0 W before an instantaneous step increase to a power output of 90% GET for 6 min (U→M). Subsequent to these 6 min of moderate‐intensity cycling, a further step increase in power output to the required severe intensity was abruptly applied, with participants exercising until they reached the limit of tolerance. During all exercise tests, pulmonary gas exchange and ventilation were measured at the mouth breath by breath with participants using a metabolic cart (Blue Cherry, Geratherm Respiratory, GmbH, Germany). Participants wore a silicone face mask (Hans Rudolph, KS,) of known dead space attached to a low‐dead space flow sensor (Geratherm Respiratory, GmbH, Germany). The metabolic cart was connected to the participant via a capillary line connected to the flow sensor. The gas analyzers were calibrated before each test using gases of known concentrations and the flow sensors were calibrated using a 3‐L syringe (Hans Rudolph, Kansas City, MO). Heart rate was measured every 1 sec during all tests using short‐range radiotelemetry (Garmin FR70, Garmin Ltd., Switzerland). For both U→S and M→S, blood was sampled from the thumb of the right hand into glass capillary tubes at rest, during the last minute of baseline pedaling preceding the exhaustive constant‐power bout, and immediately following exhaustion. Whole blood [L^−^] was determined using a Biosen lactate analyzer (Biosen C‐Line, EKF, Germany).

Throughout all experimental visits, continuous noninvasive measurements of muscle oxygenation/deoxygenation status of the *rectus femoris* (RF) and *vastus lateralis* (VL) muscles of the quadriceps were made via a frequency‐domain multidistance near‐infrared spectroscopy (NIRS) system (Oxiplex TS, ISS, Champaign, IL). The OxiplexTS uses one light‐emitting diode (LED) detector fiber bundle and eight LEDs functioning at wavelengths of 690 and 830 nm (four LEDs per wavelength). Light‐source detector separation distances of 2.25–3.75 cm for each wavelength were utilized with cell water concentration assumed constant at 70% and data sampled at 2 Hz. This NIRS device measures and incorporates the dynamic reduced scattering coefficients to provide absolute concentrations (*μ*mol L^−1^) of deoxygenated [HHb + Mb], which is relatively unaffected by changes in blood volume during exercise (De Blasi et al. 1993; Grassi et al. 2003). NIRS has been demonstrated to produce valid estimates of O_2_ extraction (De Blasi et al. 1993; DeLorey et al. 2003; Grassi et al. 2003). The absorption spectrum of [Mb] converges with that of [HHb]; therefore, at present, NIRS is unable to differentiate between the relative contributions of [Mb] and [HHb] to the overall NIRS signal (De Blasi et al. 1991; Davis and Barstow 2013). In referring to the NIRS deoxygenation signal as [HHb + Mb], the contribution of [Mb] is therefore also acknowledged. The NIRS device also provides measures of [oxygenated hemoglobin + myoglobin] ([HbO_2 _+ MbO_2_]) and [total hemoglobin + myoglobin] ([THb + Mb]) concentration (as [HbO_2 _+ MbO_2_] + [HHb + Mb]) and thus, an indication of O_2_ availability. The two flexible NIRS probes were placed longitudinally along the distal region of the VL and the mid‐belly of the RF muscle. The area underneath the probe was cleaned, shaved, and marked with washable pen such that the probe position could be replicated for each subsequent visit. The probe was held firmly in place by elastic Velcro strapping. Following each trial, depressions of the probe on the participant's skin were examined to confirm that the probe did not move during the trial, which was the case for every exercise transition. The NIRS probe was calibrated prior to each testing session using a calibration block of known absorption and scattering coefficients. Calibration was then verified using a second block of known but distinctly different absorption and scattering coefficients. Each of these procedures was according to the manufacturer's recommendations.

**Figure 1:**
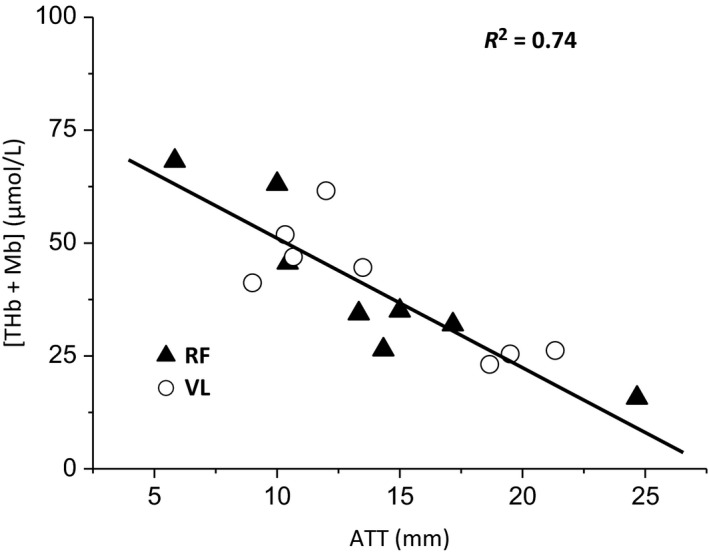
Group relationship between subcutaneous adipose tissue thickness (ATT) and resting total hemoglobin ([THb + Mb]) measured by NIRS in the rectus femoris (RF) and vastus lateralis (VL). Normalization procedure is detailed in the Methods section.

To account for the influence of adipose tissue thickness (ATT) on the NIRS signal (Koga et al. 2011) and to enable comparisons between measurement sites, we employed the correction factor used by Bowen et al. (2013). The ATT (in millimeters) was determined at each muscle site using skinfold calipers (Harpenden Skinfold Caliper, Baty International, UK) with participants standing in an upright position. Resting [THb + Mb] was then determined from a 2‐min resting average, with participants lying flat on the Echo Cardiac Stress table and strapped into the ergometric unit, with their right leg fully extended. A linear regression was then applied to the group relationship between [THb + Mb] and ATT ([THb + Mb] = −2.66 (ATT) + 77.60; *R* ^2 ^= 0.74; *P *<0.001, Fig. 1). The measured ATT at each muscle site was then substituted into the regression equation to obtain a “corrected” value of [THb + Mb], and a correction factor was then obtained by expressing the *y*‐intercept (77.60 *μ*mol/L) as a proportion of the corrected value of [THb + Mb]. The corrected [HHb + Mb], [HbO_2_ +MbO_2_], and [THb + Mb] values in all subsequent tests were then calculated as the product of the correction factor and the measured value at each site.

### Data analysis

The raw breath‐by‐breath $\dot{V}O_{2}$ data from each constant‐power exercise bout were first examined to identify data points lying more than 4 SD outside of the local mean determined using a five‐breath moving average (i.e., those data points deemed atypical of the underlying response due to coughs, sighs, swallows, etc.). Edited $\dot{V}O_{2}$ data were then subsequently linearly interpolated to produce second‐by‐second values. For $\dot{V}O_{2}$ responses to U→M, the four identical transitions were time aligned and ensemble averaged to produce a single dataset; whereas the severe‐intensity criterion bouts for determination of critical power and W’ in each condition were not repeated, therefore each were modeled separately. For each exercise transition, all data points prior to an abrupt decrease in respiratory exchange ratio and end‐tidal O_2_ pressure were excluded to remove the phase I (cardiodynamic phase) component (Whipp and Ward 1990). A single exponential function (Equation (1)) with time delay was then fitted to the data:

(1) $$\dot{V}O_{2(t)}=\dot{V}O_{2(b)}+A_{{\mathrm{vo}}_{2}}*\left(1-e^{-(t-{\mathrm{TD}}_{{\mathrm{VO}}_{2}}/\tau_{\underline{v}o_{2}})}\right),$$

where $\dot{V}O_{2(t)}$ is the $\dot{V}O_{2}$ at any time *t*; $\dot{V}O_{2(b)}$ is the baseline $\dot{V}O_{2}$ which was taken as the mean $\dot{V}O_{2}$ from the last 30 sec of the baseline cycling period preceding the exercise bout, and $A_{{\dot{V}}_{O_{2}}}$_,_ ${\text{TD}}_{{\dot{V}}_{O_{2}}}$, and $\tau_{\dot{v}o_{2}}$ are the amplitude, time delay, and time constant of the fundamental response, respectively. For moderate exercise (i.e., during U → M), Equation (1) was fitted to 360 sec. For severe exercise, the onset of the $\dot{V}O_{2}$ slow component (${\text{TD}}_{{{\mathrm{SC}V}_{O}}_{2}}$) was determined using purpose‐designed programming in Microsoft Excel (Microsoft Corporation, Redmond, WA) which fits a single exponential function to the $\dot{V}O_{2}$ data, starting at 60 sec and then iteratively extending the fitting window until the window encompasses the entire response. The resultant $\tau_{\dot{v}o_{2}}$ values are plotted against time and the ${\text{TD}}_{{{\mathrm{SC}V}_{O}}_{2}}$ was verified against the following criteria: (1) the point at which $\tau_{\dot{v}o_{2}}$ demonstrates a sustained increase from a previously “flat” profile, and (2) the demonstration of a local threshold in the *X* ^*2*^ value (Rossiter et al. 2001). This method allows the fitting of Equation (1) to the isolated fundamental phase of the $\dot{V}O_{2}$ response before the slow component can be discerned, thereby circumventing the possibility of including the slow component in the modeled fit for the fundamental phase of $\dot{V}O_{2}$. The isolated fundamental $\dot{V}O_{2}$ responses were then fitted with Equation (1) using Origin 6.0 (OriginLab Corporation, MA) to obtain the 95% confidence intervals (CIs) for the derived parameter estimates. The $\dot{V}O_{2}$ slow‐component amplitude was determined by calculating the difference between the end exercise $\dot{V}O_{2}$ (i.e., mean $\dot{V}O_{2}$ over final 30 sec of exercise) and $A_{{\dot{V}}_{O_{2}}}$ + $\dot{V}O_{2(b)}$. In instances where exercise duration was too short to allow the emergence of a slow component to be reliably discerned (typically at WR 3 and 4 in M→S and WR 4 in U→S), the $\dot{V}O_{2}$ response was modeled using Equation (1) to the end of exercise and the slow component was assigned a value of 0. The functional gain of the fundamental $\dot{V}O_{2}$ response was also calculated by dividing $A_{{\dot{V}}_{O_{2}}}$ by the change in work rate (i.e., $A_{{\dot{V}}_{O_{2}}}\Delta /\text{work rate}$ ).

The NIRS‐derived ATT‐corrected [HHb + Mb] responses to exercise were also modeled to provide information on the kinetics of muscle deoxygenation for each severe‐intensity exercise transition at both muscle sites. [HHb + Mb] increases after a short delay following the onset of step exercise, therefore the time delay before the onset of the exponential increase in [HHb + Mb] (i.e., TD_[HHb + Mb]_) was taken as the point at which [HHb + Mb] rose by 1 SD above the mean baseline value measured during the final 30 sec of baseline cycling (DeLorey et al. 2003). In instances where [HHb + Mb] decreased after the exercise onset, TD_[HHb + Mb]_ was taken as the first point following the nadir demonstrating a sustained rise in [HHb + Mb]. Data prior to this point were excluded from the model, and the [HHb + Mb] data were then fit with a monoexponential function (Equation (2)) of the form:

(2) $$\begin{array}{cc} & {\left[\text{HHb + Mb}\right]}_{\left(t\right)}={\left[\text{HHb + Mb}\right]}_{\left(b\right)}\\ & +A_{\left[\mathrm{HHb}+\mathrm{Mb}\right]}*\left(1-e^{\left(t-{\mathrm{TD}}_{\left[\mathrm{HHb}+\mathrm{Mb}\right]}\right)/\tau_{\left[\mathrm{HHb}+\mathrm{Mb}\right]}}\right) \end{array}$$

where [HHb + Mb]_*(b)*_ is the mean [HHb + Mb] measured over the final 30 sec of baseline cycling, and *A*_[HHb + Mb]_, TD_[HHb + Mb]_, and *τ*_[HHb + Mb]_ are the amplitude, time delay, and the time constant for the “fundamental” phase of the response, respectively. The model fitting window was restricted to ${\text{TD}}_{{{\mathrm{SC}V}_{O}}_{2}}$. The amplitude of the [HHb + Mb] “slow component” was calculated by subtracting the mean [HHb + Mb] measured during the final 30 sec of exercise from the absolute [HHb + Mb] response (i.e., [HHb + Mb]_*(b) *_+*A*_[HHb + Mb]_). To indicate changes in muscle oxygenation and total blood volume, the mean values for ATT‐corrected [HbO_2 _+ Mb] and [THb + Mb] were determined at baseline (30 sec prior to each transition), at 30 and 120 sec into the exercise transition (15 sec bins centered on 30 and 120 sec), and at end exercise (final 30 sec) to allow comparisons between conditions. These distinct time points were chosen to allow comparisons between conditions early in the transition during the fundamental rise of $\dot{V}O_{2}$ before the $\dot{V}O_{2}$ slow component had begun to develop, and after the $\dot{V}O_{2}$ slow component had developed fully (i.e., at the limit of tolerance). In addition to modeling the $\dot{V}O_{2}$ and [HHb + Mb] responses to exercise, we also calculated the ratio of change in [HHb + Mb] to change in $\dot{V}O_{2}$ (Δ[HHb + Mb]/Δ$\dot{V}O_{2}$) to provide further insight into the degree of reliance on O_2_ extraction to satisfy a given $\dot{V}O_{2}$ in each exercise transition. First, the second‐by‐second [HHb + Mb] data from the RF and VL for like transitions were ensemble averaged to produce a single dataset for each transition. The second‐by‐second $\dot{V}O_{2}$ and [HHb + Mb] were linearly interpolated to give 5 sec averages, and these 5‐sec averaged data were normalized for each transition (from 0%, reflecting the baseline value, to 100% reflecting the final amplitude of the response). The normalized $\dot{V}O_{2}$ data were then left shifted by the duration of phase I (which had been previously determined) so that the fundamental phase $\dot{V}O_{2}$ increase was aligned with exercise onset. A mean value of the Δ[HHb + Mb]/Δ$\dot{V}O_{2}$ ratio was calculated for each transition as the average ratio value across the duration of the fundamental phase. Intersite coefficient of variation (CV% = 100*SD/ mean of the two sites) for each participant was calculated to quantify the spatial heterogeneity for the TD_[HHb + Mb]_ and *τ*_[HHb + Mb]_.

Heart rate kinetics were modeled using a monoexponential function (Equation (3)) with the response constrained to the start of exercise (at *t *=* *0; i.e., with no time delay):

(3) $${\text{HR}}_{\left(t\right)}={\text{HR}}_{\left(b\right)}+A_{\mathrm{HR}}*\left(1-e^{\left(t/\tau_{\mathrm{HR}}\right)}\right)$$

where HR_*(b)*_ is the mean HR measured over the final 30 sec of baseline cycling, and *A*_HR_ and *τ*_HR_ are the amplitude and the time constant of the response, respectively. The fitting window was restricted to ${\text{TD}}_{{{\mathrm{SC}V}_{O}}_{2}}$.

Critical power and W’ were estimated using three mathematically equivalent models: the hyperbolic power–time (P‐T) model (Equation (4)); the linear work–time (W‐T) model, where the total work done is plotted against time (Equation (5)); and the linear inverse‐of‐time (1/T) model (Equation (6)), where power output is plotted against the inverse of time:

(4) $$P=W^{\prime }/T+\text{CP}$$

(5) $$W=\text{CP}*\text{T +}\;W^{\prime }$$

(6) $$P=W^{\prime }\left(1/T\right)*+\text{CP}$$

The standard errors of the estimates (SEE) associated with critical power and W’ were expressed as a coefficient of variation (CV) relative to the parameter estimate. The model with the lowest average CV for each participant was then used for all subsequent analyses. The same model was used in both conditions for each individual participant.

### Statistical analyses

A one‐way repeated measures ANOVA was used to compare differences in $\dot{V}O_{2}$ peak between the constant work‐rate trials and the ramp incremental test. Two‐way repeated measures ANOVAs (condition * work rate) were used to compare differences in $\dot{V}O_{2}$ and heart rate kinetic parameters, Δ[HHb + Mb]/Δ$\dot{V}O_{2}$ (data were averaged across muscle sites due to no differences between muscle sites), and the CV% for [HHb + Mb] kinetic parameters across muscle sites. Three‐way repeated measures ANOVAs (condition * work rate * time and condition * muscle * work rate) were used to compare differences in blood [L^−^] and [HHb + Mb] kinetic parameters between trials, respectively. Four‐way repeated measures ANOVAs (condition * muscle * work rate * time) were used to compare differences in [HbO_2_ + MbO_2_] and [THb +Mb] between trials. Planned repeated and simple contrasts were used to locate any significant main or interaction effects. Mauchly's test was used to test for the assumption of sphericity for repeated measures factors. Where this assumption was violated, the Greenhouse–Geisser correction factor was applied to adjust the degrees of freedom. Student's paired *t* tests were used to compare differences in critical power and W’ between conditions. Pearson's product–moment correlation coefficient was used to assess the relationship between critical power and $\tau_{\dot{v}o_{2}}$. All data are presented as mean ± SD unless otherwise stated, and 95% CI are presented for modeled time‐constant parameters. For clarity, and to highlight values for parameters measured across all four severe‐intensity work rates, the overall mean across work rates ± SD is presented in text, with work rate‐specific mean ± SD presented in tables. Statistical significance was accepted at *P* < 0.05.

## Results

The $\dot{V}O_{2}\text{max}$ and peak work rate measured during the incremental ramp test were 2.67 ± 0.44 L min^−1^ (33.0 ± 5.5 mL kg min^−1^) and 244 ± 43 W, respectively. The GET occurred at a work rate of 104 ± 15 W (1.59 ± 0.18 L min^−1^), and as such, the U→M transitions at 90% GET were conducted at a work rate of 91 ± 14 W. There was no significant main effect of condition (*P *=* *0.254) or work rate (*P *=* *0.167) on blood [L^−^], however, there was a significant main effect of time (*P *<* *0.001). Planned repeated contrasts revealed that blood [L^−^] did not differ between rest and baseline (U→S rest = 1.54 ± 0.57 mmol L^−1^ vs. U→S baseline =1.41 ± 0.44 mmol L^−1^; M→S rest = 1.65 ± 0.55 mmol L^−1^ vs. M→S baseline = 1.53 ± 0.50 mmol L^−1^), but was significantly greater at end exercise versus baseline (U→S end exercise = 9.30 ± 2.16 mmol L^−1^; M→S end exercise = 9.66 ± 2.23 mmol L^−1^). The lack of a condition * time interaction effect on blood [L^−^] (*P *=* *0.371) indicates that the desired moderate exercise intensity was achieved during the baseline in M→S, and therefore blood [L^−^] accumulation did not differ between conditions during the severe‐intensity exercise bouts. There was also no difference in $\dot{V}O_{2}$ peak between conditions (*P *=* *0.18) or compared to $\dot{V}O_{2}$ max determined in the preliminary incremental exercise test (*P *=* *0.23, Table 1).

**Table 1:** Pulmonary oxygen uptake responses to severe‐intensity constant work‐rate exercise in each condition

Parameter	U→S	M→S
$\dot{V}O_{2}$ baseline, L min^−1^ a		
WR 1	0.76 ± 0.11	1.58 ± 0.11
WR 2	0.88 ± 0.32	1.52 ± 0.10
WR 3	0.82 ± 0.11	1.45 ± 0.29
WR 4	0.80 ± 0.11	1.43 ± 0.28
${\text{TD}}_{{{\mathrm{SC}V}_{O}}_{2}}$, s		
WR 1	13 ± 10	12 ± 14
WR 2	9 ± 7	8 ± 4
WR 3	11 ± 7	7 ± 9
WR 4	11 ± 6	6 ± 9
$\tau_{\dot{v}o_{2}}$, s a		
WR 1	43 ± 15	69 ± 39
WR 2	48 ± 15	67 ± 23
WR 3	43 ± 14	74 ± 33
WR 4	46 ± 15	66 ± 20
$A_{{\dot{V}}_{O_{2}}}$, L min^−1^ a		
WR 1	1.49 ± 0.52	0.84 ± 0.43
WR 2	1.56 ± 0.59	1.03 ± 0.40
WR 3	1.69 ± 0.57	1.31 ± 0.52
WR 4	1.86 ± 0.48	1.48 ± 0.42
Absolute $A_{{\dot{V}}_{O_{2}}}$ , L min^−1^ a		
WR 1	2.26 ± 0.52	2.42 ± 0.42
WR 2	2.41 ± 0.56	2.55 ± 0.42
WR 3	2.55 ± 0.48	2.68 ± 0.48^b^
WR 4	2.66 ± 0.43	2.71 ± 0.51^b^
Gain, mL min^−1^ W^−1^ a		
WR 1	8.89 ± 0.77	10.35 ± 4.64
WR 2	8.41 ± 1.27	10.13 ± 1.52
WR 3	8.33 ± 1.31	10.86 ± 2.61
WR 4	8.57 ± 1.15	10.88 ± 1.36
${\text{SC}}_{V_{O_{2}}}$, L min^−1^ a		
WR 1	0.37 ± 0.09	0.21 ± 0.16
WR 2	0.27 ± 0.23	0.14 ± 0.15
WR 3	0.15 ± 0.13	0.04 ± 0.10
WR 4	0.06 ± 0.11	0.00 ± 0.00
End‐exercise $\dot{V}O_{2}$ (L min^−1^)		
WR 1	2.62 ± 0.57	2.63 ± 0.55
WR 2	2.71 ± 0.61	2.69 ± 0.50
WR 3	2.66 ± 0.54	2.68 ± 0.48
WR 4	2.72 ± 0.40	2.71 ± 0.51

${\text{TD}}_{V_{O_{2}}}$, fundamental time delay; $\tau_{\dot{v}o_{2}}$, fundamental time constant; $\tau_{\dot{v}o_{2}}$ 95% CI, 95% confidence interval associated with the fundamental time constant; $A_{{\dot{V}}_{O_{2}}}$ , fundamental amplitude; Absolute $A_{{\dot{V}}_{O_{2}}}$, baseline $\dot{V}O_{2}$ + fundamental $\dot{V}O_{2}$ amplitude; Gain, increase in fundamental phase $\dot{V}O_{2}$ per unit increase in power output; ${\text{SC}}_{V_{O_{2}}}$, magnitude of the $\dot{V}O_{2}$ slow component.

a Indicates significant main effect of condition,

Indicates significant main effect of work rate (*P *<* *0.05).

b Absolute amplitude of the $\dot{V}O_{2}$ response for WR 3 and 4 in M→S exceeded the value measured at end exercise, therefore, the end exercise $\dot{V}O_{2}$ is presented.

**Figure 2:**
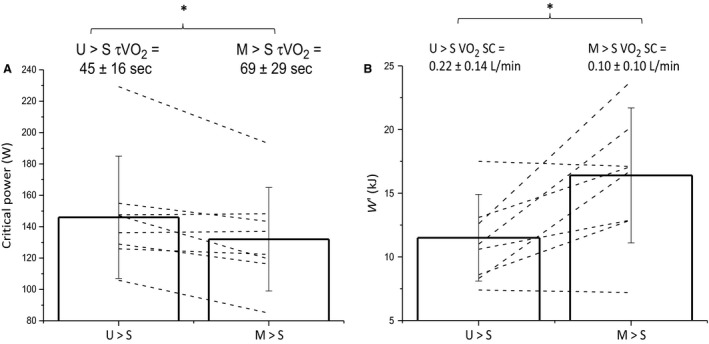
Group mean ± SD critical power (A) and *W*’ (B) in the unloaded baseline (U→S) and elevated baseline conditions (M→S). The group mean ± SD phase II $\dot{V}O_{2}$ time constant ($\tau_{\dot{v}o_{2}}$) and slow component (SC) are overlaid on panels (A) and (B), respectively. Open bars represent group means, whereas dashed black lines represent individual changes in critical power and *W*’ between conditions. * indicates significant difference between conditions (*P *<* *0.05).

**Figure 3:**
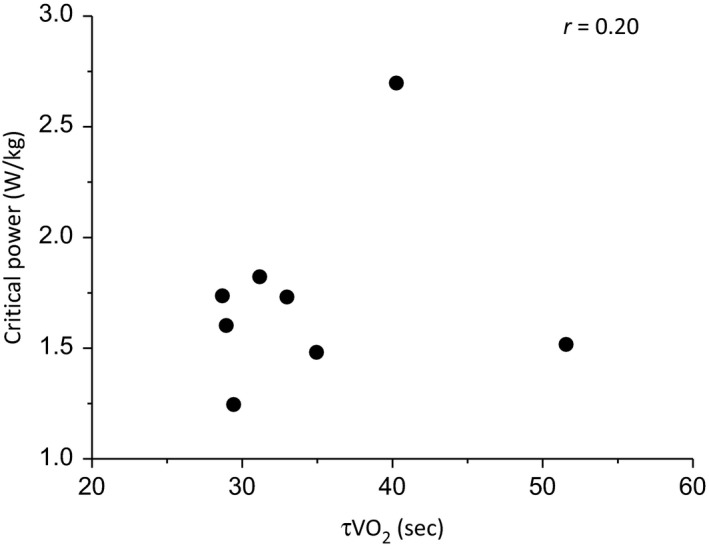
Relationship between critical power when normalized to body mass and the time constant of phase II oxygen uptake kinetics ($\tau_{\dot{v}o_{2}}$). The correlation was not significant (*P *>* *0.05).

The *W‐T* model resulted in the lowest CV for all participants, and was thus used for all subsequent analyses. Critical power was lower in M→S when compared to U→S (U→S = 146 ± 39 W, CV = 4 ± 2% vs. M→S = 132 ± 33 W, CV* *=* *5 ± 3%; *P *=* *0.023) (Fig. 2A), whereas *W*’ was greater in M→S compared to U→S (U→S = 11.5 ± 3.4 kJ, CV = 16 ± 9% vs. M→S = 16.4 ±5.3 kJ, CV = 14 ± 5%; *P *=* *0.016) (Fig. 2B). The difference in critical power between M→S and U→S was inversely correlated with the difference in W’ between M→S and U→S (*R* ^2^ = 0.92; *P *<* *0.001). $\tau_{\dot{v}o_{2}}$ in U→M was 36 ± 8 sec (95% CI, 3 ± 1 sec), and was not significantly related to critical power (normalized to body mass; determined in U→S: *r* ^^= 0.20; *P *=* *0.64, Fig. 3).

**Figure 4:**
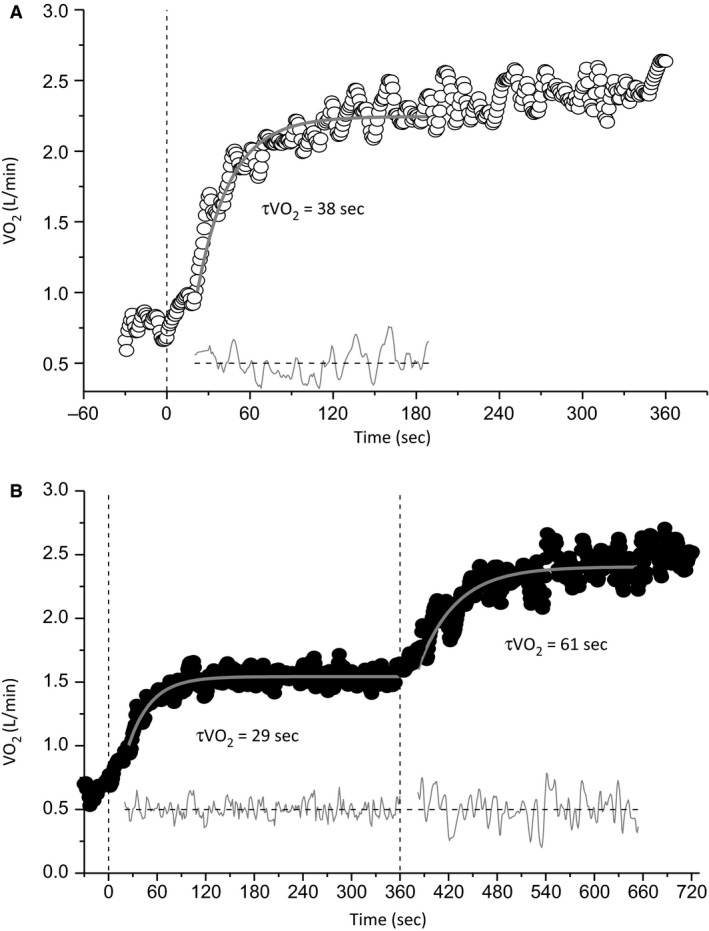
Pulmonary oxygen uptake ($\dot{V}O_{2}$) responses and best‐fit modeled responses of a representative participant in the unloaded baseline (A; U→S) and elevated baseline (B; M→S) conditions. Vertical dashed black lines represent the onset of each step transition. $\tau_{\dot{v}o_{2}}$ values are displayed for each transition, with the thick gray lines representing the modeled fits. Lines of residuals are displayed at the bottom in gray.

The $\dot{V}O_{2}$ responses of a typical participant at a representative work rate in U→S and M→S, as well as the corresponding modeled fits, are shown in Figure 4. The group mean ± SD $\dot{V}O_{2}$ kinetic parameters at each work rate for both conditions are presented in Table 1. The elevated baseline work rate resulted in the baseline $\dot{V}O_{2}$ being higher in M→S relative to U→S (U→S =0.82 ± 0.17 L min^−1^ vs. M→S = 1.49 ± 0.20 L min^−1^; *P *< 0.001), and thus the corresponding $A_{{\dot{V}}_{O_{2}}}$ was smaller (U→S = 1.65 ±0.54 L min^−1^ vs. M→S = 1.17 ±0.44 L min^−1^; *P *<* *0.001). However, the absolute amplitude (baseline + $A_{{\dot{V}}_{O_{2}}}$) of the fundamental component (U→S = 2.48 ± 0.53 L min^−1^ vs. M→S = 2.69 ± 0.55L min^−1^; *P *= 0.001) and the gain of the fundamental $\dot{V}O_{2}$ response (U→S = 8.55 ± 1.13 mL min W^−1^ vs. M→S = 10.55 ± 2.53 mL min W^−1^; *P *=* *0.05) were both greater in M→S compared to U→S. $\tau_{\dot{v}o_{2}}$ was greater in M→S compared to U→S (U→S = 45 ± 16 sec, 95% CI, 4 ± 1 sec, vs. M→S = 69 ± 29 sec, 95% CI 7 ± 2 sec; *P *=* *0.001), whereas the magnitude of the $\dot{V}O_{2}$ slow component was smaller (U→S = 0.22 ± 0.14 L min^−1^ vs. M→S = 0.10 ± 0.10 L min^−1^; *P *<* *0.001) and its onset was later in exercise (U→S = 163 ± 36 sec, vs. M→S = 212 ± 9 sec; *P *<* *0.001) in M→S compared to U→S. There was no difference in $\dot{V}O_{2}$ peak during the constant work‐rate trials between conditions (*P *=* *0.18) or work rates (*P *=* *0.49), with no interaction effect (*P *=* *0.14). Furthermore, there were no differences between $\dot{V}O_{2}$ peak measured during the constant work‐rate trials and the $\dot{V}O_{2}\text{max}$ determined in the ramp incremental test (*P *=* *0.23, Table 1).

**Figure 5:**
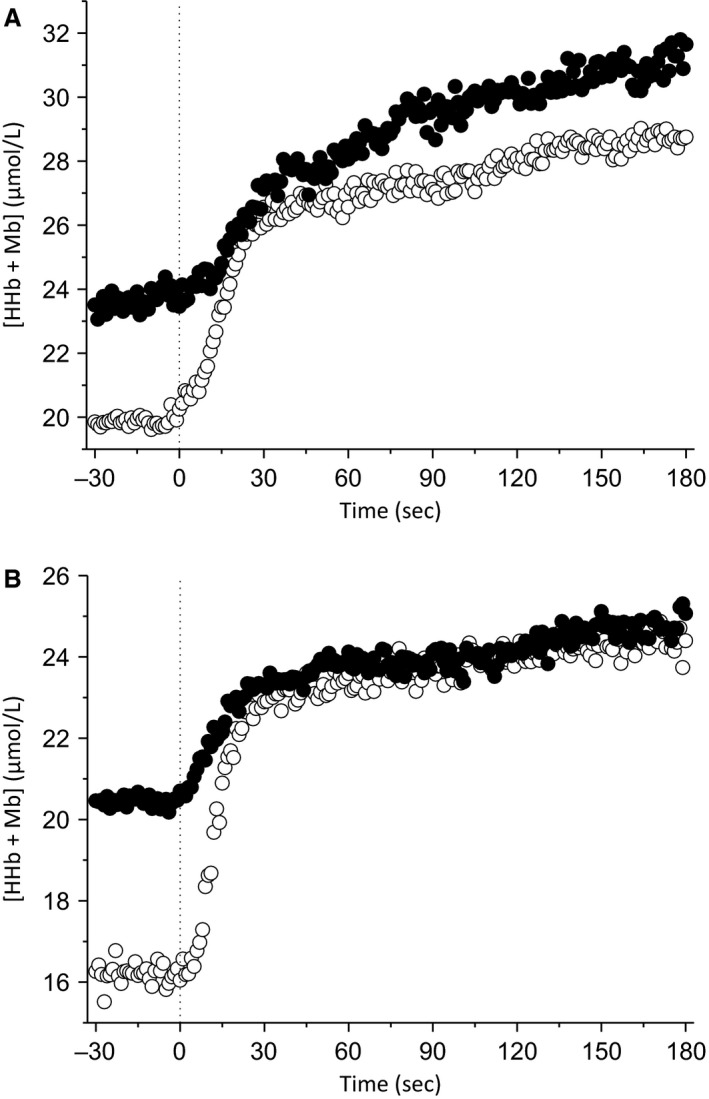
Group mean muscle deoxyhemoglobin + myoglobin ([HHb + Mb]) responses and averaged modeled fits to exercise at a single work rate for both muscle sites in the unloaded baseline (open circles) and elevated baseline (black circles) conditions. Responses for the rectus femoris (RF) are displayed in (A), whereas vastus lateralis is displayed in (B). The vertical dashed black line represents the onset of exercise.

**Figure 6:**
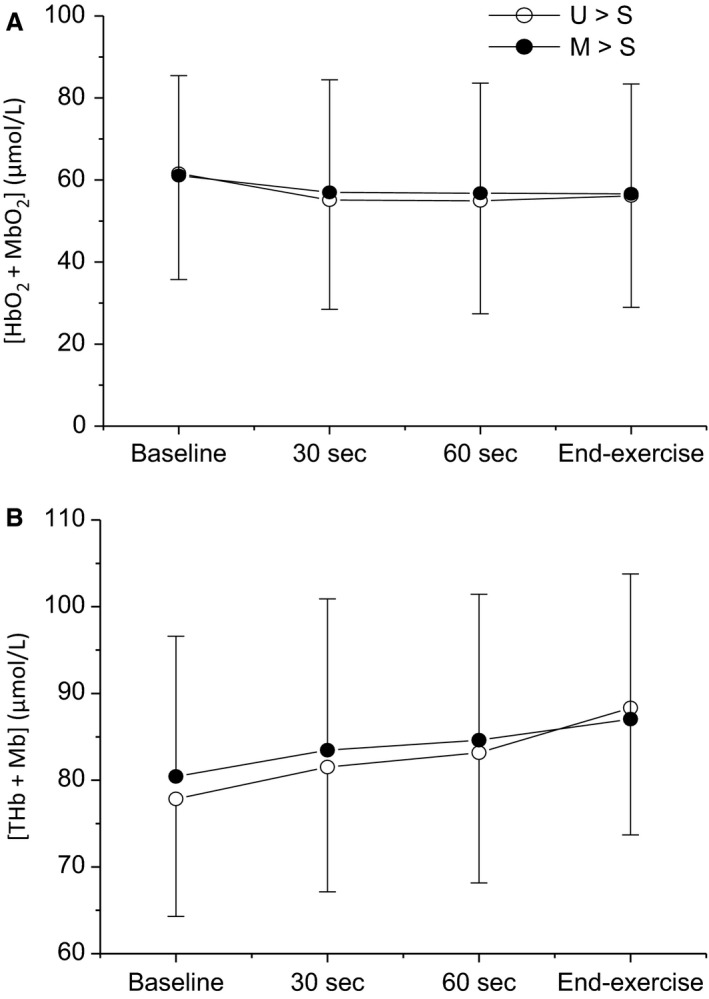
Comparisons of group means ± SD across all work rates and muscle sites for oxyhemoglobin (A; [HbO_2_  + MbO_2_]) and total hemoglobin (B; [THb + Mb]) between conditions. Open circles, unloaded baseline condition (U→S); closed circles, elevated baseline condition, (M→S). No significant differences were observed between conditions for either variable (*P *>* *0.05).

The group mean [HHb + Mb] responses to exercise at a representative work rate in each condition and both muscle sites are depicted in Figure 5. Group mean ± SD [HHb + Mb] kinetic parameters at each work rate for both conditions and muscle sites are presented in Table 2. The group mean [HbO_2_ + MbO_2_] and [THb + Mb] responses across work rates at each muscle site in both conditions are shown in Figure 6. There were no differences in [HbO_2_ + MbO_2_] (*P *=* *0.78; Fig. 6A) or [THb + Mb] (*P *=* *0.89; Fig. 6B) between muscle groups or conditions ([HbO_2_ + MbO_2_]: *P *=* *0.296; Figure 6A; [THb + Mb]: *P *=* *0.341; Fig. 6B). There were also no significant condition * time ([HbO_2_ + MbO_2_]: *P *=* *0.13; [THb + Mb]: *P *=* *0.23) or condition * time * muscle ([HbO_2_ + MbO_2_]: *P *=* *0.45; [THb + Mb]: *P *=* *0.52) interaction effects. These findings remained consistent when [HbO_2_ + MbO_2_] and [THb + Mb] for each muscle were analyzed separately in order to reduce the number of factors in the statistical analysis.

**Table 2:** Muscle deoxygenation kinetic responses at each power output for both muscle sites in M→S and U→S

	Rectus femoris		Vastus lateralis	
Parameter	U→S	M→S	U→S	M→S
[HHb + Mb]_(b)_, *μ*mol/L a				
WR 1	19.5 ± 7.1	25.7 ± 10.9	18.9 ± 6.8	19.5 ± 14.9
WR 2	19.5 ± 6.8	23.6 ± 9.6	14.2 ± 9.9	18.2 ± 10.3
WR 3	19.2 ± 8.9	24.4 ± 14.9	13.8 ± 7.8	19.0 ± 12.8
WR 4	18.1 ± 7.5	24.3 ± 11.8	11.3 ± 8.1	20.0 ± 9.3
TD_[HHb + Mb]_, s				
WR 1	6 ± 7	19 ± 38	4 ± 4	37 ± 40
WR 2	8 ± 4	7 ± 6	7 ± 7	5 ± 4
WR 3	6 ± 5	15 ± 27	7 ± 4	13 ± 24
WR 4	6 ± 3	10 ± 7	6 ± 3	27 ± 48
*τ*_[HHb + Mb]_, s a				
WR 1	15 ± 8	55 ± 38	11 ± 3	38 ± 19
WR 2	17 ± 7	61 ± 35	12 ± 6	30 ± 11
WR 3	18 ± 8	35 ± 17	11 ± 4	32 ± 29
WR 4	21 ± 11	42 ± 28	12 ± 9	22 ± 17
*A*_[HHb + Mb]_, *μ*mol L^−1^ a				
WR 1	7.5 ± 3.7	7.5 ± 5.5	10.5 ± 7.7	6.3 ± 4.6
WR 2	7.9 ± 3.6	8.2 ± 6.2	8.3 ± 6.6	7.1 ± 7.9
WR 3	9.8 ± 5.0	4.4 ± 2.6	8.5 ± 7.2	5.5 ± 3.7
WR 4	8.4 ± 3.4	7.1 ± 4.9	10.6 ± 8.4	4.8 ± 3.1
Absolute *A*_[HHb + Mb]_, *μ*mol L^−1^ a				
WR 1	27.0 ± 10.3	32.2 ± 15.2	29.4 ± 13.6	25.8 ± 18.8
WR 2	27.4 ± 10.3	31.8 ± 13.8	22.6 ± 15.8	24.4 ± 16.4
WR 3	29.1 ± 13.5	26.1 ± 15.8 b	22.3 ± 14.6	24.5 ± 13.8
WR 4	26.5 ± 10.7	30.7 ± 15.3 b	23.7 ± 16.4	24.8 ± 12.0
SC_[HHb + Mb]_, *μ*mol L^−1^				
WR 1	4.0 ± 2.7	2.3 ± 2.7	3.3 ± 2.3	0.0 ± 2.1
WR 2	2.7 ± 2.0	1.2 ± 1.3	4.1 ± 8.5	2.0 ± 3.3
WR 3	1.4 ± 1.7	0.0 ± 0.0	2.4 ± 3.0	0.0 ± 1.4
WR 4	0.7 ± 1.2	0.0 ± 0.0	0.6 ± 0.6	0.1 ± 0.6
End‐exercise [HHb + Mb] (*μ*mol L^−1^)				
WR 1	31.0 ± 11.8	34.5 ± 16.4	32.7 ± 14.5	25.8 ± 18.8
WR 2	30.1 ± 11.6	33.0 ± 13.5	26.7 ± 16.8	26.4 ± 18.4
WR 3	30.5 ± 14.3	26.1 ± 15.8	24.7 ± 14.3	24.5 ± 13.3
WR 4	27.2 ± 10.9	30.7 ± 15.3	24.3 ± 16.4	24.9 ± 12.4
Δ[HHb + Mb]/$\dot{V}O_{2}$ a				
WR 1	1.16 ± 0.18	0.82 ± 0.09		
WR 2	1.05 ± 0.21	0.95 ± 0.21		
WR 3	1.09 ± 0.20	0.85 ± 0.39		
WR 4	1.21 ± 0.27	1.02 ± 0.28		

[HHb + Mb]_(b)_, mean [HHb + Mb] over last 30 sec of baseline; TD_[HHb + Mb]_, time delay before exponential rise in [HHb + Mb]; *τ*_[HHb + Mb]_, time constant of [HHb + Mb] response; *A*_[HHb + Mb]_, amplitude of [HHb + Mb] response; Absolute *A*_[HHb + Mb]_, baseline [HHb + Mb] + amplitude [HHb + Mb]; SC_[HHb + Mb]_, magnitude of the [HHb + Mb] slow component; End‐exercise [HHb + Mb], mean [HHb + Mb] during finals 30 s of exercise; Δ[HHb + Mb]/$\dot{V}O_{2}$, ratio of Δ[HHb + Mb] to Δ$\dot{V}O_{2}$, values presented as means across muscles.

a Indicates significant main effect of condition (*P *<* *0.05).

b Absolute amplitude of the [HHb + Mb] response for WR 3 and 4 in M→S exceeded the value measured at end exercise, therefore, the end‐exercise [HHb + Mb] is presented. There was no main effect of condition on TD_[HHb + Mb]_, however, in four participants for a total of seven exercise transitions in M→S, there was a long period (i.e., >60 sec) of no change in [HHb + Mb] followed by an exponential increase, thus accounting for the relatively larger mean TD_[HHb + Mb]_ and SD values in M→S.

Baseline [HHb + Mb] was greater in M→S than in U→S (RF U→S = 18.1 ± 7.5 *μ*mol/L vs. RF M→S = 24.3 ± 11.8 *μ*mol/L; VL U→S = 15.4 ± 7.7 *μ*mol/L vs. VL M→S = 18.8 ± 10.9 *μ*mol/L; *P *=* *0.044), whereas *A*_[HHb + Mb]_ (RF U→S = 8.4 ± 3.4 *μ*mol/L vs. RF M→S = 7.1 ± 4.9 *μ*mol/L; VL U→S = 9.6 ± 7.1 *μ*mol/L vs. VL M→S = 5.8 ± 4.2 *μ*mol/L; *P *=* *0.015) and the resulting Δ[HHb + Mb]/Δ$\dot{V}O_{2}$ (U→S = 1.14 ± 0.13 vs. M→S = 0.91 ± 0.11; *P *=* *0.046) ratio were decreased in M→S compared to U→S. Furthermore, *τ*_[HHb + Mb]_ was greater in M→S when compared to U→S (RF U→S = 18 ± 7 sec, 95% CI 2 ± 1 sec vs. RF M→S =48 ± 16 sec, 95% CI 8 ± 2 sec; VL U→S = 11 ± 4 sec, 95% CI 1 ± 1 sec vs. VL M→S = 30 ± 11 sec, 95% CI 6 ± 5 sec; *P *<* *0.001). The lack of significant main effects of muscle (all *P *>* *0.05) or any condition * muscle interaction effects (all *P *>* *0.05) indicated that these effects were consistent across the muscle sites. There was no significant difference in the spatial heterogeneity (assessed as the between‐site CV) of the *τ*_[HHb + Mb]_ (U→S =28 ± 24% vs. M→S = 46 ± 27%; *P *=* *0.92) or TD_[HHb + Mb]_ (U→S = 38 ± 47% vs. M→S = 45 ±28%; *P *=* *0.22) between conditions.

Baseline heart rate was greater in M→S compared to U→S (U→S = 87 ± 10 beats min^−1^ vs. M→S =119 ± 13 beats min^−1^; *P *=* *0.034), however, *τ*_HR_ was not significantly different between conditions (U→S =46 ± 20 sec, 95% CI 3 ± 2 sec, vs. M→S = 68 ± 41 sec, 95% CI 6 ± 5 sec; *P *=* *0.11).

## Discussion

The primary purpose of this study was to test the hypothesis that $\tau_{\dot{v}o_{2}}$ is not a primary determinant of critical power in conditions when O_2_ delivery is limiting to $\tau_{\dot{v}o_{2}}$. In accordance with our first hypothesis, $\tau_{\dot{v}o_{2}}$ was increased during exercise initiated from an elevated baseline work rate (M→S) compared to an unloaded baseline work rate (U→S). However, the novel finding of this study was that, in contrast to our second hypothesis, there was a concomitant reduction in critical power in M→S.

The present findings are of significance because they add to a growing body of evidence suggesting that the fundamental time constant of muscle $\dot{V}O_{2}$ ($\dot{V}O_{2m}$) kinetics, determined via proxy measurement at the lung, that is, $\tau_{\dot{v}o_{2}}$, is an independent determinant of critical power. Significantly, as we have previously demonstrated this phenomenon during exercise transitions that are not limited by O_2_ availability, that is, upright cycle exercise in young healthy adults (Goulding et al. 2018), the present data suggest that $\tau_{\dot{v}o_{2}}$ is an independent determinant of critical power, irrespective of the sufficiency of O_2_ availability. Indeed, the present data suggest that our previous finding of an increased critical power following priming exercise in the supine position (Goulding et al. 2017) was indeed due to a reduced $\tau_{\dot{v}o_{2}}$, rather than the attendant improvement in O_2_ availability. Although this study was undertaken in young healthy individuals, our data may also have implications for understanding the etiology of impaired exercise tolerance in clinical populations that are also characterized by an O_2_ delivery limitation, such as heart failure (Hirai et al. 2015) and diabetes (Behnke et al. 2002).

Central to the interpretation of the present data is the influence of a raised baseline work rate on O_2_ availability during the transition to severe‐intensity exercise. Consistent with previous research in the upright position (Spencer et al. 2011; Breese et al. 2013; Williams et al. 2013) we observed that *τ*_[HHb + Mb]_ was greater in M→S when compared to U→S, reflecting slower muscle deoxygenation kinetics in the field of interrogation. This could be due to an excess O_2_ availability, relative to the O_2_ demand, as indicated by the lower Δ[HHb + Mb]/Δ$\dot{V}O_{2}$ ratio in M→S. However, absolute indicators of O_2_ availability, [THb + Mb] and [HbO_2_ + MbO_2_], were unchanged between conditions at any time, including early in the exercise transition (Fig. 6). This suggests that the slower muscle deoxygenation kinetics and lower Δ[HHb + Mb]/Δ$\dot{V}O_{2}$ ratio were due to the profound slowing of pulmonary $\dot{V}O_{2}$ kinetics (and by implication, $\dot{V}O_{2\;m}$ kinetics), and not due to an enhancement of O_2_ availability, during the exercise transition in M→S compared to U→S.

Despite this interpretation, NIRS instrumentation does not provide a measurement of muscle blood flow. Hence, the performance of prior exercise may have improved O_2_ delivery hemodynamics via a greater red blood cell velocity and increased longitudinal recruitment of capillaries (Hudlická et al. 1982; Kindig et al. 2002), with no difference in underlying tissue concentrations of HbO_2_. An improved $\dot{V}O_{2}$ /muscle O_2_ delivery ($\dot{Q}O_{2}$) ratio during the criterion exercise transition would allow the same $\dot{V}O_{2}$ to be achieved with a lower rate of O_2_ extraction, and thus alternatively explain the greater *τ*_[HHb + Mb]_ and lower Δ[HHb + Mb]/Δ$\dot{V}O_{2}$ ratio in M→S. However, according to the size principle (Henneman and Mendell 1981) work‐to‐work exercise results in the recruitment of a “new” pool of higher‐order motor units that were not previously contributing to force production (Brittain et al. 2001). As these higher‐order, ostensibly type II motor units muscle fibers are poorly vascularized compared to their type I counterparts (Crow and Kushmerick 1982; Willis and Jackman 1994; Reggiani et al. 1997) and were not previously active, it is difficult to envisage any hemodynamic improvements in O_2_ delivery occurring as a result of prior exercise. Importantly, we also observed no difference between conditions in the CV for either *τ*_[HHb + Mb]_ or TD_[HHb + Mb]_, suggesting that the initiation of exercise from an elevated baseline work rate did not exacerbate any existing regional limitations in O_2_ delivery or heterogeneities in the $\dot{V}O_{2}$/$\dot{Q}O_{2}$ ratio. Thus, the present data support previous research indicating that the increase in $\tau_{\dot{v}o_{2}}$ due to work‐to‐work exercise is intramuscular in origin, and likely due to the recruitment of a greater proportion of type II fibers possessing inherently slower $\dot{V}O_{2}$ kinetics (DiMenna et al. 2010a) and/or the reduced cellular energetic state in previously contracting muscle fibers (i.e., *P*O_2_ and [PCr], increased [ADP] and [P_i_], and less negative Gibbs free energy for ATP hydrolysis) (Meyer and Foley 1996).

### $\tau_{\dot{v}o_{2}}$–critical power relationship

Critical power represents the upper limit of the physiological steady state during exercise, with continued exercise above critical power, even during constant work‐rate exercise, resulting in the attainment of $\dot{V}O_{2}\text{max}$ (Poole et al. 1988). Given that the results of this study implicate $\tau_{\dot{v}o_{2}}$ as a determinant of this effect, independent of O_2_ availability, we propose the mechanistic basis explaining this effect likely relates to the intracellular drivers of oxidative phosphorylation. During the transition to high‐intensity exercise, the initial rise in intracellular [ADP] is a primary driver of increases in oxidative metabolism via a relationship that has been shown to be sigmoidal in vivo (Wüst et al. 2011). As exercise progresses, the large O_2_ deficit invoked by high‐intensity exercise and corresponding accumulation of metabolic byproducts associated with anaerobic metabolism will result in the fatigue of already recruited fibers (Cannon et al. 2011) and/or the progressive recruitment of higher‐order muscle fibers (Krustrup et al. 2008). Hence, [ADP] and the O_2_ cost of exercise will progressively rise, with [ADP] moving further toward the plateau region of the [ADP]–$\dot{V}O_{2}$ curve, desensitizing the $\dot{V}O_{2}$ response to further elevations in [ADP]. Whether the $\dot{V}O_{2}$ response will plateau therefore rests upon whether the rise in $\dot{V}O_{2}$ stimulated by further increases in [ADP] is sufficient to match the demands for ATP turnover, or if a progressive reliance on anaerobic metabolism occurs. The former reflects the attainment of a physiological steady state whereas the latter would result in a cascade of fatigue‐related physiological events, ultimately resulting in the attainment of $\dot{V}O_{2}$ max and exercise intolerance (Poole et al. 1988; Jones et al. 2008; Murgatroyd et al. 2011). We have therefore previously proposed that critical power represents the work rate at which a “critical” [ADP] is attained during the rest–exercise transition, above which there is an inability to attain a metabolic steady state due to a lack of sensitivity of $\dot{V}O_{2}$ to rising [ADP] (Goulding et al. 2018). Hence, $\tau_{\dot{v}o_{2}}$ is an independent determinant of critical power, as a smaller $\tau_{\dot{v}o_{2}}$ would abate the intracellular perturbation in [ADP] during the exercise transition, thus enabling a higher work rate (i.e., critical power) to be attained before a critical [ADP] is achieved. Our present data suggest that this mechanism is also important during exercise transitions limited by O_2_ availability.

This proposed mechanism is also likely to explain the strong inverse relationship between critical power and $\tau_{\dot{v}o_{2}}$ during upright cycle exercise in young, healthy individuals (Murgatroyd et al. 2011; Goulding et al. 2017, 2018). However, the present (Fig. 3) and our previous (Goulding et al. 2017) data show that this relationship is absent during supine exercise, despite the present data suggesting that $\tau_{\dot{v}o_{2}}$ is an independent determinant of critical power in this body position. This apparent contradiction could be explained by the relationship between $\tau_{\dot{v}o_{2}}$ and critical power being curvilinear (i.e., hyperbolic, sigmoidal, or an alternative, more complex function) rather than linear. The previously observed relationship (Murgatroyd et al. 2011a; Goulding et al. 2017, 2018) may have appeared linear because the $\tau_{\dot{v}o_{2}}$ values only covered the range where the relationship is well approximated by a linear function. However, our data in the supine position (Goulding et al. 2017; Fig. 2) are within this “linear” range of $\tau_{\dot{v}o_{2}}$ values (Murgatroyd et al. 2011, Rossiter 2011). An alternative possibility therefore is that the resting perfusion conditions during supine exercise (i.e., loss of the hydrostatic gradient, thus lowering the pressure head for blood‐to‐myocyte O_2_ diffusion (Koga et al. 1999)) may have introduced a kinetic dissociation between pulmonary and $\dot{V}O_{2\;m}$ (Benson et al. 2013). Under such conditions of low resting perfusion, therefore, the venous O_2_ content draining the muscle (Cv_O2 *m*_) would undershoot the steady‐state response, and have the effect of distorting the pulmonary $\dot{V}O_{2}$ relative to $\dot{V}O_{2}\;m$, such that pulmonary $\dot{V}O_{2}$ would express faster kinetics than $\dot{V}O_{2\;m}$ (Benson et al. 2013). The reliance on pulmonary $\dot{V}O_{2}$ kinetics as a proxy for $\dot{V}O_{2\;m}$ kinetics in the supine position could thus be obscuring the relationship with critical power in this body position.

### Effect of baseline work rate on *W*’

In this study, we observed a ~5 kJ increase in *W*’ when exercise was initiated from an elevated baseline work rate compared to a baseline of unloaded cycling. The present data resemble previous studies demonstrating that interventions which increase critical power tend to reduce *W*’ (Vanhatalo et al. 2010), and vice versa (Broxterman et al. 2015), although such a reciprocal relationship is not ubiquitous (Jones et al. 2003; Burnley et al. 2011). Nevertheless, a reduction in critical power with an unchanged maximal $\dot{V}O_{2}$, as in this study, would increase the applicable intensity range of the power–duration relationship, such that the range of work rates which would draw upon the finite *W’* is extended (Vanhatalo et al. 2010). Such an interpretation would account for an increase in *W’* in this study and is supported by the observation of a strong inverse correlation between the change in critical power and *W*’ induced by work‐to‐work exercise (*R* ^2^ = 0.92).

The $\dot{V}O_{2}$ slow component has previously been proposed to be a determinant of *W*’, on the basis of the observation of a positive relationship between the amplitude of the $\dot{V}O_{2}$ slow component and the *W*’ (Murgatroyd et al. 2011). However, in this study we observed a decreased amplitude of the $\dot{V}O_{2}$ slow component alongside the increased *W*’ in M→S, similar to that observed following priming exercise (Jones et al. 2003; Burnley et al. 2011). The progressive recruitment of type II, supplementary to type I, muscle fibers is, at least in part, considered to be responsible for the development of the $\dot{V}O_{2}$ slow component (Krustrup et al. 2008). Hence, the reduced slow‐component amplitude during M→S may result from a $\dot{V}O_{2}$ response that reflects the metabolic properties of a more homogenous (i.e., predominantly type II) group of muscle fibers (Wilkerson and Jones 2006). The $\dot{V}O_{2}$ slow component per se does therefore not appear to be a determinant of W’. Rather, the previously observed relationship between the slow component and *W*’ (Murgatroyd et al. 2011) likely reflects the temporal development of fatigue‐related processes that only happen progressively above critical power (i.e., progressive recruitment of type II fibers, reduced P/O ratio and/or contractile efficiency in recruited fibers, and O_2_ cost of recovery in fatigued fibers) and ultimately result in task failure and thus determine the limits of W’. These processes ultimately result in task failure and thus determine the limits of *W*’. Interventions that alter the predominant contributions of these processes toward the expression of the $\dot{V}O_{2}$ slow component will thus remove its relationship with *W*’.

### Methodological considerations

Due to the demands on participants within the study, we were unable to undertake repeated visits to improve the signal‐to‐noise ratio in the $\dot{V}O_{2}$ data for severe exercise transitions, or improve confidence in the parameters derived from the power–duration relationship. However, the former issue was mitigated by the use of a high‐amplitude response, the increased $\tau_{\dot{v}o_{2}}$ in the supine position, and the delayed onset of the slow component in M→S. Consequently, the 95% CI associated with the $\tau_{\dot{v}o_{2}}$ was ~5.5 sec; which is close to the recently suggested minimally important difference to determine significant changes in intervention studies (Benson et al. 2017) and smaller than the mean difference in $\tau_{\dot{v}o_{2}}$ at all four work rates. Additionally, the mean CV was ~5% and ~15% for critical power and *W*’, respectively, both of which are smaller than the change between conditions in each parameter. We are therefore confident in the reported differences in the power–duration relationship between conditions. Despite our confidence in the main effect of work‐to‐work exercise on $\tau_{\dot{v}o_{2}}$, the relative lack of precision inherent when modeling single exercise transitions reduces confidence in the precise value for $\tau_{\dot{v}o_{2}}$. Consequently, undertaking a comparison of $\Delta \tau_{\dot{v}o_{2}}$ and Δ critical power for examining the potential causal relationship between $\tau_{\dot{v}o_{2}}$ and critical power is highly problematic. There is also no justification for any particular work rate being most representative of the underlying $\dot{V}O_{2\;m}$ kinetics. Furthermore, the supine position may result in a dissociation between pulmonary and $\dot{V}O_{2\;m}$ kinetics (Benson et al. 2013). Taken together, we therefore felt that there was no sound basis on which to undertake comparison of $\Delta \tau_{\dot{v}o_{2}}$ and Δ critical power between conditions and thus chose not to undertake this analysis.

### Conclusions

In summary, the present data implicate $\dot{V}O_{2}$ kinetics as an independent determinant of critical power in conditions of limited O_2_ availability. Specifically, during supine exercise $\tau_{\dot{v}o_{2}}$ was greater and critical power was reduced when exercise was initiated from an elevated compared to an unloaded baseline work rate. Importantly, our data show that the existing O_2_ delivery limitation imposed through the use of supine exercise was not exacerbated or meaningfully reversed when exercise was initiated from an elevated baseline work rate. Taken together with our previous data (Goulding et al. 2017, 2018), these results therefore suggest that $\tau_{\dot{v}o_{2}}$ is an independent determinant of critical power, irrespective of the sufficiency of O_2_ availability in any given condition. The results of this study also suggest that $\tau_{\dot{v}o_{2}}$ may be an effective target for interventions aimed at improving exercise tolerance in populations where $\tau_{\dot{v}o_{2}}$ becomes limited by O_2_ delivery.

## Conflict of Interest

None declared.
