# “Work‐to‐Work” exercise slows pulmonary oxygen uptake kinetics, decreases critical power, and increases W’ during supine cycling

**DOI:** 10.14814/phy2.14020

**Published:** 2019-02-19

**Authors:** Richie P. Goulding, Denise M. Roche, Simon Marwood

Physiol Rep, 6 (21), 2018, e13916, https://doi.org/10.14814/phy2.13916

In Richie et al. (2018), the following error was published in the Abstract:

The line (U→S = 45 ± 16 sec, vs. M→S = 69 ± 129 sec, *P* = 0.001) is incorrect and should instead be (U→S = 45 ± 16 sec, vs. M→S = 69 ± 29 sec, *P* = 0.001).

The authors apologise for the error.
